# The role of gut microbiota in prostate cancer progression: A Mendelian randomization study of immune mediation

**DOI:** 10.1097/MD.0000000000038825

**Published:** 2024-07-05

**Authors:** Song Li, Ruoxuan Liu, Xuexue Hao, Xiaoqiang Liu

**Affiliations:** a Department of Urology, Tianjin Medical University General Hospital, Tianjin, China; b Department of Urology, Huaihe Hospital of Henan University, Kaifeng, China; c Plastic Surgery Department, Henan International Joint Laboratory of Cellular Medical Engineering, Henan, China.

**Keywords:** gut microbiota, Mendelian randomization, prostate cancer, Tregs

## Abstract

The potential relationship between the gut microbiota and prostate cancer, possibly influenced by immune cells, remains unclear. This study employed the mediation Mendelian randomization (MR) technique to investigate the causal link between the gut microbiota, immune cells, and prostate cancer. Data on immune cell activity were sourced from Valeria Orrù’s research, whereas the genome-wide association study outcome dataset was obtained from the Integrative Epidemiology Unit database. The bidirectional MR analysis utilized 5 different methods: inverse variance weighted (IVW), weighted median, MR-Egger regression, weighted mode, and simple mode. In addition, the mediating effect of immune cells on the gut microbiota and prostate cancer was explored using mediation analysis. Eighty-three single nucleotide polymorphisms associated with prostate cancer were screened as instrumental variables. In a positive MR analysis with gut microbiota as the exposure factor, IVW showed an association between 8 gut microbiota and prostate cancer. Additionally, 9 types of immune cells have been found to be associated with prostate cancer using methods such as IVW. MR analysis of the gut microbiota on immune cells (beta1) revealed a negative correlation between Bifidobacterium and CD39^+^ T regulatory cells (Tregs; odds ratio [OR] = 0.785, 95% confidence interval [CI] = 0.627–0.983, *P* = .03). Furthermore, MR analysis of immune cells in prostate cancer disease (beta2) showed that CD39^+^Tregs are a risk factor for prostate cancer (OR = 1.215, 95% CI = 1.027–1.354, *P* = .04). Moreover, MR analysis of gut microbiota in prostate cancer (total effect) indicated that Bifidobacterium is a protective factor for prostate cancer (OR = 0.905, 95% CI = 0.822–0.977, *P* = .04). The sensitivity analysis verified the robustness of the above results. Mediation analysis demonstrated that CD39^+^Tregs partially mediate the causal relationship between Bifidobacterium and prostate cancer. This study demonstrates that Bifidobacterium inhibits prostate cancer progression through CD39^+^Tregs as mediators, providing new ideas and approaches for the treatment and prevention of prostate cancer.

## 1. Introduction

Prostate cancer is one of the most common tumors threatening men’s health, and it has the fastest increase in incidence rate at present.^[1]^ Among the relative risk factors for prostate cancer, dietary habits account for an important proportion, and men who frequently consume foods containing high animal fat are at high risk for prostate cancer.^[2]^ From the research results of 32 countries, it was found that the incidence rate of people who eat a diet rich in vegetables and fruits was low. Changes in the composition of gut microbiota can affect dietary compounds and nutrient metabolites related to the risk of prostate cancer.^[3]^ With the development of 16S rRNA sequencing technology, research on gut microbiota has become a hot topic in recent years.

Many tumors (such as ovarian cancer, sarcoma, and melanoma), although not directly in contact with the gut microbiota, are also regulated by the gut microbiota during their progression. In mice with melanoma, a high-fat diet can lead to dysbiosis of the gut microbiota, manifested by an increase in Clostridium, which can induce tumor infiltration of melanoma by activating nuclear factor kappa-B signaling in macrophages.^[4]^ In sarcoma, TLR5 recognizes the gut microbiota and promotes myeloid-derived suppressor cell mobilization, thereby accelerating tumor growth. In addition, gut microbiota can promote ovarian cancer progression by promoting interleukin-17A.^[5]^ With increasing research on the regulation of gut microbiota in the body’s response to immunotherapy, new possibilities have been provided for improving tumor efficacy through targeted regulation of gut microbiota in the future.^[6]^ However, there is currently limited research on the relationship between gut microbiota, immune microenvironment, and prostate cancer, and most of these are observational studies, which are often limited in their ability to demonstrate causal hypotheses due to confounding factors, reverse causality, and other biases. Mendelian randomization (MR) has been a widely used causal inference method in the field of epidemiology in recent years. By introducing genetic variation as instrumental variables (IVs) to analyze the causal relationship between exposure factors and outcomes, biases related to environmental exposure factors, and behavioral, social, psychological, and other factors, the problem of reverse causal relationships can be solved. This is a higher level of evidence than that of traditional observational epidemiological studies and randomized controlled studies.^[7]^ To explore the causal relationship between gut microbiota, immune microenvironment, and prostate cancer and to avoid the influence of confounding factors and reverse causality, this study used the MR method to analyze the causal relationship between gut microbiota, immune cells, and prostate cancer, providing a basis for the prevention and treatment of prostate cancer.

## 2. Materials and methods

### 2.1. Data sources

The summary data of gut microbiota were obtained from the Microbiome Genome (MiBioGen) Alliance, which included 24 cohorts and recruited 18,340 study subjects from different races.^[8]^ The immune cell–related data comes from Valeria Orrù study, which performed a genome-wide association study (GWAS) on 272 blood immune cell–related traits profiles by flow cytometry in 1629 general population individuals.^[9]^ The latest GWAS exposure summary data with the largest sample size were obtained from the Integrative Epidemiology Unit database (http://docs.epigraphdb.org/research-studies/ieu-gwas-database/). The GWAS data for prostate cancer incidence risk includes 211,227 patients and 199,628 control groups,^[10]^ as shown in Table 1.

**Table 1:** Details of the genome-wide association studies and datasets used in our analyses.

Exposure or outcome	Sample size	Ancestry	Links for data download	PMID
Human gut microbiome	18,340 participants	Mixed	https://mibiogen.gcc.rug.nl	33462485
Prostate cancer	211,227 cases, 199,628 control	European	https://gwas.mrcieu.ac.uk/datasets/ebi-a-GCST90018905/	34594039
22 million variants on 731 immune cell traits	3757 participants	European	https://doi.org/10.1038/s41588-020-0684-4	32929287

### 2.2. Selection of IVs

First, single nucleotide polymorphisms (SNPs) highly associated with prostate cancer from the GWAS database of gut microbiota (exposure) were selected (*P* < 1 × 10^−5^). Then, according to the criterion of linkage disequilibrium, SNPs with *r*^2^ < 0.001 were retained to ensure independence between IVs. Next, we removed SNPs directly associated with the outcome (prostate cancer) (*P* < 5 × 10^−8^). For the excluded SNPs, using the European population in the Thousand Genome Project as a reference standard, alternative SNPs with *r*^2^ > 0.8 under the linkage disequilibrium criterion. SNPs with *F* < 10 were excluded to eliminate bias caused by weak IVs.^[11]^ Finally, 83 SNPs were used as IVs (Supplementary Table 1, Supplemental Digital Content, http://links.lww.com/MD/N127).

### 2.3. Mendelian randomization

Multiple analysis methods were used to investigate the causal relationship between exposure factors and prostate cancer, including inverse variance weighted (IVW), weighted median (WME), MR-Egger regression, weighted mode, and simple mode. The results of IVW were used as the main basis, whereas other methods were used as a supplementary basis and validation for IVW. The odds ratio (OR) and 95% confidence interval (CI) were calculated using univariate MR analysis, and sensitivity analysis was performed. Cochran’s *Q* test was used to test for heterogeneity among IVs, with a *P* value less than .05 indicating heterogeneity. MR-Egger regression was used to verify pleiotropy, and leave-one-out analysis was also conducted to determine the degree of influence of a single SNP on causal relationships.

### 2.4. Reverse MR analysis

To determine the direction of causal association and exclude the influence of reverse causal association on the results, this study conducted reverse MR analysis. Reverse MR analysis was used to evaluate the causal relationship between prostate cancer as the exposure factor and gut microbiota as the outcome factor. When the forward MR analysis has statistical significance, while the reverse MR analysis has no statistical significance, it can further confirm the direction of the causal effects.

### 2.5. Mediation analysis

**Figure 1.**
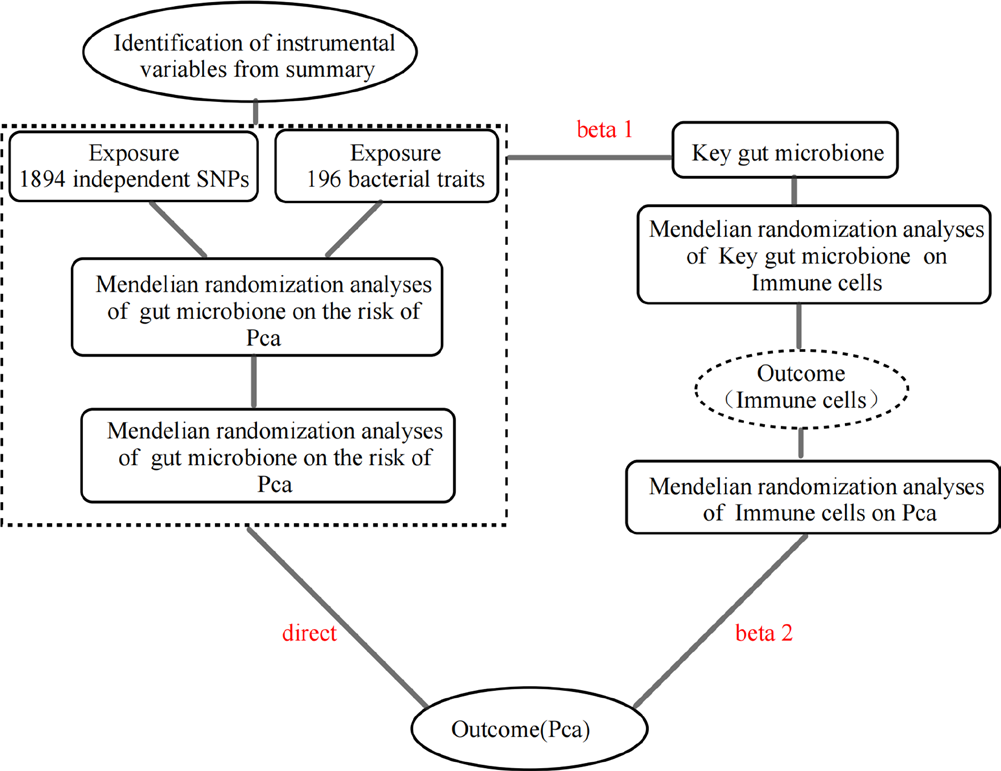
Design for mediated Mendelian randomization. Pca = prostate cancer, SNPs = single nucleotide polymorphisms.

We conducted a mediation analysis using 2-step MR to investigate whether immune cells mediate causal pathways from the gut microbiota on prostate cancer outcomes. The overall effect can be decomposed into indirect (through a medium) and direct effects (without a medium). Specifically, this includes MR randomization analysis of the effect of gut microbiota on immune cells (beta1), MR randomization analysis of the effect of immune cells on prostate cancer (beta2), and calculation of the mediating effect (beta1 × beta2) and direct effect (total effect-mediating effect), as shown in Figure 1.

### 2.6. Statistical analysis

Forest maps were drawn using the “grid,” “reader,” and “forestploter” of the R software package. All statistical analyses in this study were performed based on the “Two-Sample MR,” “VariantAnnotation,” “gwasglue,” and “ieugwasr” package of the R software (R version 4.2.3). The evaluation indicators were OR and 95% CI, with *P* < .05 was considered statistically significant.

## 3. Results

### 3.1. Screening of gut bacteria related to prostate cancer

**Figure 2.**
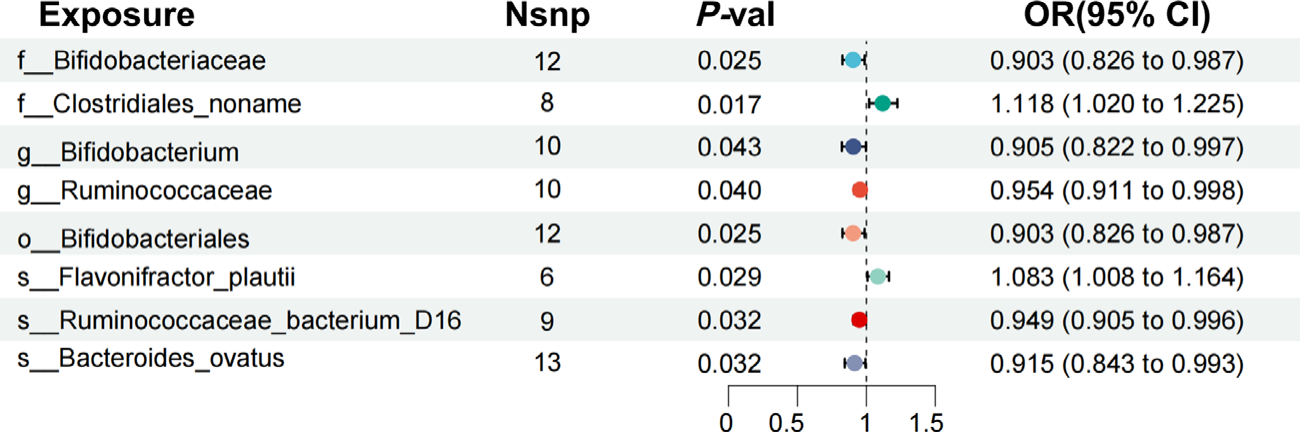
Screening of the gut microbiota associated with the risk of Pca. CI = confidence interval, nsnp = number of single nucleotide polymorphism, OR = odds ratio, Pca = prostate cancer.

In forward MR analysis, the IVW method found an association between 12 gut microbiota and prostate cancer, among which 8 gut microbiota showed relatively stable causal associations with consistent directions in the IVW, WME, and MR-Egger results. Bifidobacteriaceae, Bifidobacterium, Ruminococcaceae, Bifidobacteriales, Ruminococcaceae_bacterium_D16, and Bacteroides_ovatus are potential protective factors for prostate cancer (OR < 1, *P* < .05); Clostridiales and Flavonifractor_plautii were potential risk factors for prostate cancer (OR >1; *P* < .05), as shown in Figure 2.

### 3.2. Reverse MR analysis

This study used prostate cancer as an exposure factor and conducted reverse MR analysis with gut microbiota, showing a causal association with prostate cancer as the outcome in forward MR analysis. The MR-Egger regression results showed that the selected IVs did not exhibit pleiotropy (all *P* > .05). The use of different MR methods showed that prostate cancer was not the cause of gut microbiota at various classification levels (*P* > .05), further confirming the direction of causal effects between the gut microbiota and prostate cancer, as shown in Supplementary Table 2, Supplemental Digital Content, http://links.lww.com/MD/N128.

### 3.3. Screening immune cells related to prostate cancer

In the MR analysis, a relatively stable causal association was found between the nine types of immune cells and prostate cancer through the IVW, WME, and MR-Egger methods. Among them, Mo-myeloid-derived suppressor cell AC and granulocyte AC are potential protective factors for the onset of prostate cancer (*P* < .05). IgD^+^CD38br AC, IgD^+^CD38dim AC, TDDN (CD4-CD8-) AC, CD3 on NKT, HLADR on CD14^+^ monocytes, HVEM on EM CD4^+^, and CD39^+^ T regulatory cells (Tregs) were potential risk factors for prostate cancer (*P* < .05), as shown in Table 2.

**Table 2:** Screening of the immune cells associated with the risk of Pca.

Exposure	Outcome	Method	Nsnp	*b*	*P* value	OR
IgD^+^ CD38br AC	Pca	Inverse variance weighted	23	0.068	.002	1.070
IgD^+^ CD38dim AC	Pca	Inverse variance weighted	20	0.092	.001	1.096
Mo-MDSC AC	Pca	Inverse variance weighted	18	−0.039	.007	0.962
TDDN (CD4-CD8-) AC	Pca	Inverse variance weighted	20	0.075	.007	1.078
Granulocyte AC	Pca	Inverse variance weighted	21	−0.072	.007	0.931
CD3 on NKT	Pca	Inverse variance weighted	13	0.078	.007	1.081
HVEM on EM CD4^+^	Pca	Inverse variance weighted	19	0.051	.000	1.052
HLADR on CD14^+^ monocyte	Pca	Inverse variance weighted	19	0.042	.007	1.043
CD39^+^ Treg	Pca	Inverse variance weighted	14	0.033	.042	1.215

Pca = prostate cancer.

### 3.4. Mediation MR analysis

#### 3.4.1. MR analysis of gut microbiota to immune cells (beta1)

**Figure 3.**
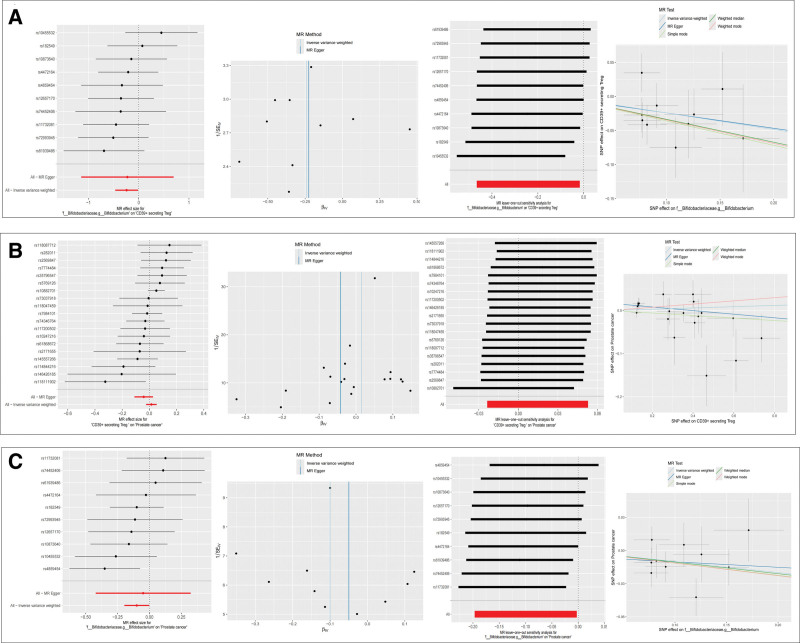
Mediation MR analysis. (A) MR effect size, funnel plot, MR leave-one-out sensitivity, and scatter plot for Bifidobacteriaceae on CD39^+^ secreting Treg. (B) MR effect size, funnel plot MR leave-one-out sensitivity, and scatter plot for CD39^+^ secreting Treg on prostate cancer. (C) MR effect size, funnel plot, MR leave-one-out sensitivity, and scatter plot for Bifidobacteriaceae on prostate cancer. MR = Mendelian randomization, SNPs = single nucleotide polymorphisms, Treg = T regulatory cells.

First, this study conducted a MR analysis of the gut microbiota in immune cells. The causal relationship between gut microbiota and immune cells was analyzed using the IVW, WME, and MR-Egger methods. The scatter plot showed a negative correlation between Bifidobacterium and CD39^+^ Treg cells, whereas the forest plot confirmed a significant negative correlation between the total effect value of SNP. The funnel plot also showed that the distribution of the estimated causal effects of the IVs was symmetrical, and there was no overall heterogeneity. In addition, the leave-one-out method was used to perform a one-by-one exclusion test on SNPs, and the estimated differences in the effects of each SNP were not statistically significant, proving the robustness of this result (Fig. 3A).

#### 3.4.2. MR analysis of immune cells to prostate cancer (beta2)

Second, we conducted MR analysis of immune cells in prostate cancer. The scatter plot indicated that CD39^+^ Treg cells were a risk factor for prostate cancer, and the forest plot also confirmed a positive correlation between the total effect value of the SNPs. The funnel plot also indicated no overall heterogeneity. The leave-one-out method was used to perform a one-by-one exclusion test on the SNPs, and there was no statistically significant difference in the estimated effects of each SNP (Fig. 3B).

#### 3.4.3. MR analysis of Bifidobacterium to prostate cancer (total effect)

Finally, we conducted MR analysis (total effect) of the gut microbiota on prostate cancer. The causal relationship between gut microbiota and prostate cancer was analyzed using the IVW, WME, and MR-Egger methods. The scatter plot suggests that Bifidobacterium is a protective factor against prostate cancer. The forest plot also confirmed that the total effect value of the SNP was significantly negatively correlated. The funnel plot also showed that the distribution of the estimated causal effects of the IVs was symmetrical, and there was no overall heterogeneity. The robustness of this result was demonstrated by performing a one-by-one exclusion test on the SNPs using the leave-one-out method (Fig. 3C).

**Figure 4.**
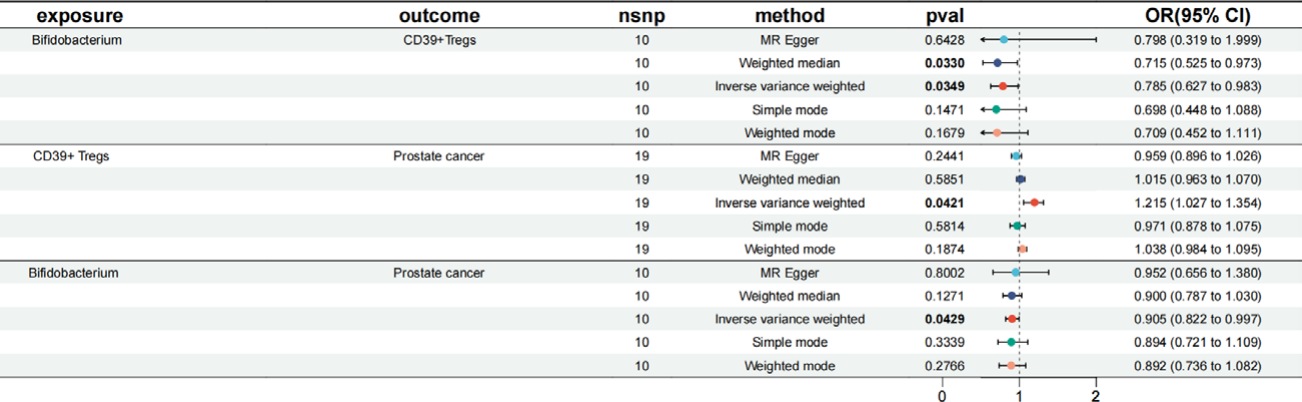
The forest plot of mediated Mendelian randomization. CI = confidence interval, MR = Mendelian randomization, nsnp = number of single nucleotide polymorphism, OR = odds ratio.

In summary, the forest plot showed that Bifidobacterium inhibited the abundance of CD39^+^Tregs using the IVW method (OR = 0.785, *P* = .0349), CD39^+^Tregs were a risk factor for prostate cancer (OR = 1.015, *P* = .0421), and Bifidobacterium was a protective factor for prostate cancer (OR = 0.905, *P* = .0429) (Fig. 4). Mediated MR analysis showed that Bifidobacterium inhibits prostate cancer progression through CD39^+^Tregs (Table 3).

**Table 3:** Mediator Mendel effect value.

Gut microbiome	Immune cell	Outcome	BetaAll	Mediated effect	Mediated proportion
Bifidobacterium	CD39^+^ Tregs	Prostate cancer	−0.11	−0.013 (−0.05, 0.05)	3.54%

## 4. Discussion

The incidence of prostate cancer is related to factors such as age, race, family history, environmental factors, immune responses, and genetics. Interestingly, there is a significant difference in the gut microbiota between patients with prostate cancer and healthy individuals or patients with benign prostate disease.^[12]^ Golombos et al^[13]^ evaluated the gut microbiota of 20 patients with benign prostatic hyperplasia and prostate cancer and found that the relative abundance of *Pseudomonas aeruginosa* was higher in the gut of prostate cancer patients, whereas the relative abundance of fecal bacteria was lower. Matsushita et al^[14]^ found that the abundance of Bacteroidetes, such as Vibrio, Streptomyces, and Spirulina, in the feces of high-risk prostate cancer patients was significantly higher than that in non-cancerous males. Liu and Jiang^[15]^ conducted a matched analysis of the fecal microbiota of 21 hormone-sensitive prostate cancer and castration-resistant prostate cancer patients who received androgen deprivation therapy and found that there were significant changes in the gut microbiota of castration-resistant prostate cancer patients, mainly manifested as an increase in the abundance of several bacterial communities, such as Ruminococcus. This is consistent with our findings. We found a stable causal association between eight gut microbiota and prostate cancer using the IVW method, among which Bifidobacterium, Ruminococcaceae, Bifidobacteriales, Ruminococcaceae_bacterium_D16, and Bacteroidesovatus were potential protective factors for the onset of prostate cancer; Clostridiales and Flavonifractor_plautii were potential risk factors for the development of prostate cancer. Bifidobacterium exhibits antiproliferative and apoptotic effects in human prostate cancer cell lines (PC-3 and DU-145).^[16]^ Ruminococcaceae influences Pca progression through its metabolites, long-chain fatty acids.^[17]^ However, Clostridium regulates systemic and local prostate insulin-like growth factor1 in the host body through the action of short-chain fatty acids, promoting the proliferation of prostate cancer cells.^[14]^ Lactic acid-producing bacteria, such as bifidobacteria, may be valuable in enhancing the immune response to prostate cancer and bladder cancer.^[18]^ This indicates that the gut microbiota affects prostate cancer progression through various pathways, including metabolism, inflammation, and immune responses.

Gut microbiota plays a role in mediating inflammation, immune regulation, and regulation of substance metabolism in the human body.^[14]^ The gut microbiome can affect the occurrence and development of prostate cancer through various mechanisms, among which inducing systemic chronic inflammation and regulating the immune system may be its main pathway.^[19]^ Sivan et al^[20]^ demonstrated an unexpected role for commensal Bifidobacterium in enhancing antitumor immunity in vivo. Bifidobacterium induces immune background regulation by triggering antitumor host immune responses, possibly by enhancing the biosynthesis of immune stimulatory molecules and metabolites, and synergistically reducing tumor burden with PD-1 blocker therapy.^[21]^

In recent years, studies have suggested a close relationship between gut microbiota and innate and acquired immunity in the intestine.^[22]^ Tregs are a type of immune negative regulatory cells that can inhibit the killing effect of the body’s immune system on tumor cells through immunosuppressive and immune nonfunctional effects, helping tumor cells escape the immune response and leading to rapid tumor growth and metastasis.^[23]^ The occurrence and development of prostate cancer are related to the tumor microenvironment and immune escape. Tregs inhibit the immune response of the body, construct the tumor microenvironment, and assist in tumor immune escape. This study screened 9 immune cells with relatively stable causal associations with prostate cancer through MR analysis, among which CD39^+^Tregs were a potential risk factor for prostate cancer. The correlation forest plot also confirmed a positive correlation between the total effect value of SNP, and the funnel plot also showed that there was no heterogeneity in the whole body. This suggests that CD39^+^secreting Tregs may assist tumor immune escape by inhibiting the body’s immune response, thereby promoting prostate cancer progression. Interestingly, the gut microbiota can regulate the distribution of Tregs in intestinal tissues.^[24]^ Pandiyan et al^[25]^ found that gut microbiota has a significant impact on Treg differentiation. The lack of gut microbiota can limit the differentiation of Tregs in the intestine; however, the regulatory mechanisms of the 2 are complex and are currently not fully understood. Therefore, this study conducted a MR analysis (beta1) of the gut microbiota in the immune cells. Through IVW, WME, and MR-Egger analyses, it was found that Bifidobacterium was negatively correlated with CD39^+^Tregs. Analysis of the overall effect showed that Bifidobacterium was a protective factor against prostate cancer, and there was no overall heterogeneity. The leave-one-out method was used to perform a one-by-one exclusion test on the SNPs, and the estimated effects of each SNP were not statistically significant. Bifidobacterium first acts on the intestinal mucosa, then on the intestinal and extraintestinal immune systems, inducing differentiation and migration of Treg immune cells in the intestine, and can affect the levels of systemic cytokines.^[26]^ Bifidobacterium maintains the Treg/Th17 balance in mice by downregulating interleukin-33 production and inhibiting the TLR2/4 signaling pathway.^[27]^ These previously published results are consistent with the findings described here, which demonstrated that Bifidobacterium can inhibit prostate cancer progression through CD39^+^ Treg immune cells as mediating factors.

This study adopted the MR method using SNP as an IV to effectively avoid the influence of various confounding factors in traditional epidemiological studies. However, this study has certain limitations. First, the gut microbiome data come from a mixed population, while the data on prostate cancer and immunity both come from the European population, which leads to certain differences in genetic data between different races, but still has certain reference values. Second, the threshold of our IVs (*P* value, *F* statistic) is an effect, but we did not utilize the Bonferroni-corrected method and remove false negative errors. Finally, future investments are required to employ more European populations as a source of the GWAS dataset. Future clinical trials are necessary to validate the potential pathologies, development, and treatment of prostate cancer.

## 5. Conclusions

In summary, this study used gut microbiota and immune cells as exposure factors and employed bidirectional MR and mediated MR analysis methods to explore the causal relationship between gut microbiota, immune cells, and prostate cancer. Bifidobacterium inhibits the progression of prostate cancer through CD39^+^Tregs as mediators. Therefore, future antitumor treatments can combine the gut microbiota and immunotherapy methods, providing new ideas and approaches for the treatment and prevention of prostate cancer. However, further in vitro and in vivo experiments are required to elucidate the potential biological mechanisms of this study.

## Author contributions

**Data curation:** Song Li, Ruoxuan Liu.

**Formal analysis:** Song Li.

**Funding acquisition:** Song Li, Ruoxuan Liu.

**Methodology:** Song Li, Xuexue Hao.

**Project administration:** Song Li.

**Resources:** Song Li.

**Software:** Song Li.

**Writing – original draft:** Song Li.

**Supervision:** Xiaoqiang Liu.

**Visualization:** Xiaoqiang Liu.

**Writing – review & editing:** Xiaoqiang Liu.

## Supplementary Material
