# Supplementary material for: The role of gut microbiota in prostate cancer progression: A Mendelian randomization study of immune mediation
Source: Medicine (Baltimore). 2024 Jul 5;103(27):e38825. doi: 10.1097/MD.0000000000038825 (PMC11224845; doi:10.1097/MD.0000000000038825)
Supplement: Supplementary file 1 [file medi-103-e38825-s001.docx]

**Supplementary Table 1**. Information on SNPS included in this study

| SNP | chr | pos | EA | OA | beta | pval | R2 | F |
| --- | --- | --- | --- | --- | --- | --- | --- | --- |
| rs1546579 | 1 | 61319955 | T | C | -0.077 | 7.18E-06 | 0.003 | 20.139 |
| rs182549 | 2 | 136616754 | T | C | -0.153 | 5.86E-15 | 0.008 | 60.934 |
| rs74452406 | 3 | 63012872 | T | C | -0.176 | 2.16E-07 | 0.003 | 26.879 |
| rs11732081 | 4 | 62600906 | T | C | 0.078 | 6.94E-06 | 0.003 | 20.206 |
| rs4859454 | 4 | 77541509 | T | C | 0.123 | 3.05E-06 | 0.003 | 21.778 |
| rs4472164 | 4 | 168344708 | G | A | -0.125 | 5.31E-06 | 0.003 | 20.717 |
| rs12657170 | 5 | 79643369 | C | T | 0.079 | 2.73E-06 | 0.003 | 21.992 |
| rs10455532 | 6 | 92883361 | G | A | -0.077 | 4.26E-06 | 0.003 | 21.138 |
| rs77432108 | 8 | 74446780 | T | A | 0.096 | 8.93E-06 | 0.003 | 19.722 |
| rs61939486 | 12 | 119663906 | C | T | -0.111 | 5.44E-06 | 0.003 | 20.672 |
| rs10873640 | 15 | 24025547 | T | C | 0.091 | 6.03E-06 | 0.003 | 20.475 |
| rs34823013 | 19 | 552579 | G | T | -0.087 | 7.79E-06 | 0.003 | 19.983 |
| rs315026 | 1 | 77041173 | G | A | -0.102 | 8.44E-06 | 0.003 | 19.831 |
| rs12039057 | 1 | 181338339 | G | A | -0.105 | 8.81E-06 | 0.003 | 19.749 |
| rs36131910 | 4 | 150277279 | A | G | 0.235 | 1.28E-06 | 0.003 | 23.454 |
| rs74564383 | 10 | 59135772 | T | C | -0.226 | 3.58E-06 | 0.003 | 21.469 |
| rs72896141 | 11 | 5845481 | A | G | -0.172 | 2.80E-06 | 0.003 | 21.942 |
| rs35903463 | 13 | 85599144 | T | A | 0.161 | 4.07E-06 | 0.003 | 21.228 |
| rs7402939 | 15 | 99183876 | C | T | 0.105 | 2.99E-06 | 0.003 | 21.819 |
| rs4939587 | 18 | 47142173 | G | T | -0.132 | 6.41E-07 | 0.003 | 24.779 |
| rs182549 | 2 | 136616754 | T | C | -0.152 | 8.59E-15 | 0.008 | 60.180 |
| rs74452406 | 3 | 63012872 | T | C | -0.171 | 4.61E-07 | 0.003 | 25.413 |
| rs11732081 | 4 | 62600906 | T | C | 0.077 | 8.55E-06 | 0.003 | 19.805 |
| rs4859454 | 4 | 77541509 | T | C | 0.120 | 4.54E-06 | 0.003 | 21.015 |
| rs4472164 | 4 | 168344708 | G | A | -0.126 | 4.97E-06 | 0.003 | 20.842 |
| rs12657170 | 5 | 79643369 | C | T | 0.077 | 4.10E-06 | 0.003 | 21.210 |
| rs10455532 | 6 | 92883361 | G | A | -0.077 | 4.14E-06 | 0.003 | 21.194 |
| rs61939486 | 12 | 119663906 | C | T | -0.109 | 8.28E-06 | 0.003 | 19.866 |
| rs10873640 | 15 | 24025547 | T | C | 0.091 | 6.01E-06 | 0.003 | 20.481 |
| rs72993945 | 18 | 77284465 | A | G | -0.082 | 6.67E-06 | 0.003 | 20.280 |
| rs114446285 | 2 | 127826259 | G | A | -0.200 | 3.95E-06 | 0.003 | 21.284 |
| rs12621499 | 2 | 142324536 | G | A | 0.176 | 7.35E-06 | 0.003 | 20.096 |
| rs570295 | 6 | 12732095 | G | A | 0.192 | 1.56E-07 | 0.004 | 27.507 |
| rs3916427 | 9 | 113929557 | C | T | -0.164 | 4.29E-06 | 0.003 | 21.127 |
| rs9414689 | 9 | 140215415 | G | C | 0.204 | 7.09E-06 | 0.003 | 20.164 |
| rs9508731 | 13 | 30981374 | T | A | -0.327 | 1.93E-06 | 0.003 | 22.659 |
| rs11626630 | 14 | 75207510 | T | C | 0.241 | 5.34E-06 | 0.003 | 20.705 |
| rs4416113 | 18 | 42991796 | A | G | 0.327 | 2.59E-06 | 0.003 | 22.090 |
| rs4805798 | 19 | 28473852 | A | G | 0.170 | 4.21E-06 | 0.003 | 21.159 |
| rs4239490 | 19 | 51132056 | C | T | 0.234 | 3.79E-06 | 0.003 | 21.362 |
| rs1546579 | 1 | 61319955 | T | C | -0.077 | 7.20E-06 | 0.003 | 20.135 |
| rs182549 | 2 | 136616754 | T | C | -0.153 | 5.91E-15 | 0.008 | 60.914 |
| rs74452406 | 3 | 63012872 | T | C | -0.176 | 2.18E-07 | 0.003 | 26.860 |
| rs11732081 | 4 | 62600906 | T | C | 0.078 | 6.94E-06 | 0.003 | 20.204 |
| rs4859454 | 4 | 77541509 | T | C | 0.123 | 3.05E-06 | 0.003 | 21.780 |
| rs4472164 | 4 | 168344708 | G | A | -0.125 | 5.29E-06 | 0.003 | 20.722 |
| rs12657170 | 5 | 79643369 | C | T | 0.079 | 2.72E-06 | 0.003 | 21.998 |
| rs10455532 | 6 | 92883361 | G | A | -0.077 | 4.24E-06 | 0.003 | 21.149 |
| rs77432108 | 8 | 74446780 | T | A | 0.096 | 8.88E-06 | 0.003 | 19.732 |
| rs61939486 | 12 | 119663906 | C | T | -0.111 | 5.39E-06 | 0.003 | 20.686 |
| rs10873640 | 15 | 24025547 | T | C | 0.091 | 6.02E-06 | 0.003 | 20.477 |
| rs34823013 | 19 | 552579 | G | T | -0.087 | 7.79E-06 | 0.003 | 19.984 |
| rs13061928 | 3 | 2932365 | G | A | -0.179 | 5.44E-06 | 0.003 | 20.670 |
| rs9848037 | 3 | 55796723 | G | A | 0.150 | 7.07E-06 | 0.003 | 20.168 |
| rs77544959 | 11 | 39603353 | A | G | -0.149 | 5.80E-06 | 0.003 | 20.547 |
| rs1872436 | 12 | 57322492 | T | C | -0.182 | 8.84E-07 | 0.003 | 24.159 |
| rs9513795 | 13 | 101402225 | T | C | 0.159 | 9.00E-06 | 0.003 | 19.708 |
| rs5768347 | 22 | 48492960 | G | T | -0.169 | 8.29E-06 | 0.003 | 19.865 |
| rs114446285 | 2 | 127826259 | G | A | -0.201 | 3.66E-06 | 0.003 | 21.429 |
| rs12621499 | 2 | 142324536 | G | A | 0.175 | 8.26E-06 | 0.003 | 19.871 |
| rs570295 | 6 | 12732095 | G | A | 0.191 | 1.67E-07 | 0.004 | 27.372 |
| rs3916427 | 9 | 113929557 | C | T | -0.164 | 4.30E-06 | 0.003 | 21.121 |
| rs9414689 | 9 | 140215415 | G | C | 0.205 | 6.49E-06 | 0.003 | 20.331 |
| rs9508731 | 13 | 30981374 | T | A | -0.327 | 2.02E-06 | 0.003 | 22.566 |
| rs11626630 | 14 | 75207510 | T | C | 0.241 | 5.67E-06 | 0.003 | 20.591 |
| rs4416113 | 18 | 42991796 | A | G | 0.326 | 2.61E-06 | 0.003 | 22.075 |
| rs4805013 | 19 | 28484840 | T | A | 0.168 | 4.73E-06 | 0.003 | 20.937 |
| rs4239490 | 19 | 51132056 | C | T | 0.234 | 3.59E-06 | 0.003 | 21.465 |
| rs303925 | 1 | 48503280 | T | C | -0.084 | 4.52E-06 | 0.003 | 21.025 |
| rs59809293 | 2 | 33849581 | A | C | -0.083 | 1.39E-06 | 0.003 | 23.286 |
| rs190949005 | 2 | 56319707 | G | A | -0.154 | 9.07E-06 | 0.003 | 19.692 |
| rs112057053 | 3 | 127211963 | C | G | 0.146 | 5.85E-07 | 0.003 | 24.953 |
| rs9684138 | 4 | 38287192 | T | C | -0.091 | 1.90E-06 | 0.003 | 22.691 |
| rs73240572 | 4 | 57505068 | C | T | 0.086 | 7.03E-06 | 0.003 | 20.181 |
| rs72677788 | 4 | 126550852 | A | G | -0.184 | 7.39E-07 | 0.003 | 24.504 |
| rs4867356 | 5 | 31623146 | C | T | 0.100 | 3.86E-06 | 0.003 | 21.329 |
| rs67659622 | 8 | 142862278 | A | G | 0.099 | 3.83E-07 | 0.003 | 25.772 |
| rs1021440 | 9 | 94379045 | G | A | -0.083 | 4.82E-06 | 0.003 | 20.901 |
| rs10820045 | 9 | 104830105 | A | C | -0.166 | 7.58E-06 | 0.003 | 20.036 |
| rs75642318 | 11 | 112426121 | G | A | -0.128 | 4.10E-06 | 0.003 | 21.211 |
| rs2642158 | 17 | 7630301 | G | C | 0.096 | 5.06E-07 | 0.003 | 25.234 |
| rs11873510 | 18 | 5850906 | C | T | -0.124 | 2.90E-06 | 0.003 | 21.879 |
| rs9304475 | 18 | 22785354 | T | C | -0.088 | 3.60E-07 | 0.003 | 25.892 |
