# Supplementary material for: The role of gut microbiota in prostate cancer progression: A Mendelian randomization study of immune mediation
Source: Medicine (Baltimore). 2024 Jul 5;103(27):e38825. doi: 10.1097/MD.0000000000038825 (PMC11224845; doi:10.1097/MD.0000000000038825)
Supplement: Supplementary file 2 [file medi-103-e38825-s002.docx]

**Supplementary Table 2**. Reverse Mendelian randomization analysis of gut bacteria for the Pca

| Exposure | Outcome | Method | Nsnp | b | p-value | OR |
| --- | --- | --- | --- | --- | --- | --- |
| Prostate cancer | f_Bifidobacteriaceae | Inverse variance weighted | 12 | -0.102 | 0.176 | 0.90 |
| Prostate cancer | f_Clostridiales_noname | Inverse variance weighted | 8 | 0.112 | 0.209 | 1.12 |
| Prostate cancer | g_Bifidobacterium | Inverse variance weighted | 10 | -0.100 | 0.171 | 0.90 |
| Prostate cancer | g_Ruminococcaceae_noname | Inverse variance weighted | 10 | -0.047 | 0.496 | 0.95 |
| Prostate cancer | o_Bifidobacteriales | Inverse variance weighted | 12 | -0.102 | 0.176 | 0.90 |
| Prostate cancer | s_Flavonifractor_plautii | Inverse variance weighted | 6 | 0.080 | 0.891 | 1.08 |
| Prostate cancer | s_Ruminococcaceae_bacterium_D16 | Inverse variance weighted | 9 | -0.052 | 0.622 | 0.95 |
| Prostate cancer | s_Bacteroides_ovatus | Inverse variance weighted | 13 | -0.089 | 0.198 | 0.91 |
